# Thyroid surgery in geriatric patients: a literature review

**DOI:** 10.1186/1471-2482-12-S1-S16

**Published:** 2012-11-15

**Authors:** Rita Gervasi, Giulio Orlando, Maria Antonietta Lerose, Bruno Amato, Giovanni Docimo, Pio Zeppa, Alessandro Puzziello

**Affiliations:** 1 General Surgery Unit, Dept of General Surgery, Geriatric and Endoscopy, University Federico II, Naples, Italy; 2 VII General Surgery Unit, Dept of General Surgery, Second University of Naples, Italy; 3 Endocrinosurgery Unit, Dept of Medical and Surgical Sciences, University Magna Graecia, Catanzaro, Italy

## Abstract

**Background:**

Thyroid disease is common in the elderly population. The incidence of hypothyroidism and multinodular goitre gradually increases with age. In view of a growth of aging population, we performed a literature review about the feasibility of thyroid surgery in the elderly.

**Methods:**

We conducted a literature search in the PubMed database in September 2012 and all English-language publications on thyroidectomy in geriatric patients since 2002 were retrieved. The potential original articles mainly focusing on thyroidectomy in elderly patients were all identified and full texts were obtained and reviewed for further hand data retrieving.

**Results:**

We retrieved five papers based on different primary end-point. Four were retrospective non randomized studies and one was prospective non randomized study. At last 65, 70, 75 and 80 years were used as an age cut-off. All studies evaluate the indications of thyroidectomy in geriatric patients, postoperative morbility and mortality. Only one study specifically assesses the rate of the rehospitalization after thyroidectomy among the elderly.

**Conclusions:**

Thyroid nodules are particularly important in elderly patients, as the incidence of malignancy increases and they are usually more aggressive tumors. An age of at least 70 years is an independent risk factor for complications after general surgery procedures. Thyroid surgery in patients aged 70 years or older is safe and the relatively high rate of thyroid carcinoma and toxic goiter may justify an aggressive approach. A programmed operation with a careful pre-operative evaluation and a risk stratification should make the surgical procedures less hazardous, specially in 80 years old patients with an high ASA score.

## Introduction

In 2000 in the world there were about 600 million people with more than 60 years, in 2025 there will be 1.2 billion and 2 billion in 2050. People who survive to the ages of 70 to 75 years may be expected to live 14 additional years; those who live to ages of 80 to 85 years, 8 additional years.

However, an exact definition of the geriatric patient is not available in the medical literature [1,2]. Various publications differ in the age defined, which may be 60, 65 or 70 years; there are even studies placing it around 80 years [3].

On the other hand the prevalence of nodular thyroid conditions increases considerably with age; 90% of women present with thyroid nodules after the age of 60 years, and 60% of men after the age of 80 years. Almost 50% of patients ≥65 years demonstrate nodules on ultrasound examination, with a similar prevalence among autopsies performed for the general population [4,5].

Therefore, we performed this review of global literature to provide current state-of-the-art data on the number and type of the publications, the indications, the clinical outcomes in thyroid surgery in geriatric patients.

## Material and methods

The literature search was conducted in the PubMed database in September 2012, and all English-language publications on CE since 2002 were retrieved. The search terms that we selected were ‘‘thyroidectomy or thyroid surgery or thyroid disease and elderly or older adult or geriatric patient’’ which were mainly based on the official thesaurus (MeSH). All initial search results were reviewed by title and abstracts. Then, the potential original articles mainly focusing on CE were all identified, and full texts were obtained and reviewed for further hand data retrieving.

## Results

We selected only five papers with different primary end-point. Four were retrospective non randomized studies and three of them with a younger control group; one was prospective non randomized study of consecutive patients undergoing thyroidectomy by a single surgeon. At last 65, 70, 75 and 80 years were used as an age cut-off. All studies evaluate the indications of thyroidectomy in geriatric patients, postoperative morbility and mortality.

The type of studies and the reviewed data are reported in Table 1 and 2.

**Table 1:** Type of studies on thyroidectomy in elderly patients

Study	Type of study	Age cut-off	Number of elderly patient	Median age	Post operative malignancy	Total/subtotal thyroidectomy
Passler[1]	Case-control retrospective	75	55/738	79.9	36.4% (vs 26.2%) [p NS]	90.9%

Rios[3]	Case-control retrospective	65	81/510	72	Not reported	79%

Mekel[4]	Case-control retrospective	80	90/242	83.2	20% (vs 27%) [p NS]	60%

Seybt[5]	Prospective non randomized	65	44/86	71.3	27.3% (vs 18.6%)	100%

Raffaelli[10]	Retrospective	70	320	73.3	26.6%	88.4%

**Table 2:** Early and permanent complications

Study	ASA score >III	Early postoperative complications	Permanent complications	
	
			Hypoparathyroidism	Paralysis of RLN
Passler[1]	Not reported	25.5% (21.8%) [p NS]	2.3% (vs 2.0%)	1.05% (vs 0.26%)

Rios[3]	18%	37% (vs 21%)	0% (vs 1.6%)	2.5% (vs 2.4%)

Mekel[4]	45.2%	Not reported*	Not reported*	Not reported*

Seybt[5]	Not reported	15.4% (vs 15.0%)	0% (vs 0%)	0% (vs 0%)

Raffaelli[10]	16.6%	34.7%	1.6%	0.2%

RLN – recurrent laryngeal nerve

* the study reports thyroid specific complications including 1% readmission for hypocalcemia and 1% vocal cord dysfunction in the octogenarians and 2% vocal cord dysfunction and 0.4% hematomas in the control group.

Raffaelli et al. [6] report 320 consecutive patients with a median age of 73.3 years without a control group and a rate of performed total thyroidectomy of 88,4% (Table 1). The rate of early postoperative complications (hypoparathyroidism and recurrent laryngeal nerve palsy) is similar to the other studies (Table 2), except Seybt et al [5] (15.4%) where the age cut-off is 65 years so that more younger patients are included in elderly group.

The most feared complication of thyroid surgery, recurrent nerve palsy, has a rate ranging from 0% (Seybt et al) [5] to 2.5% (Rios et al) [3] without a significant difference with the younger group. Furthermore Seybt et al [5] report a 44 consecutive patients undergoing total thyroidectomy by a single surgeon as the younger group, without a permanent complications.

## Discussion

In our study, we found no differences between the aged and the younger groups with regard to postoperative morbidity and mortality. The p value, when reported, was non-significant and this suggests the feasibility and safety of the thyroid surgery in elderly.

On the other hand Mekel [4] found that the postoperative complication rates increased significantly with age from nearly 9% in the control group (mean age 50,1 yr) to >24% in the octogenarians.

It is important to note that the high complication rate in patients over the age of 80 yr, as reported in Mekel [4]; was not due to the usually observed post-thyroidectomy complications, such as nerve injury, hypoparathyroidism, or post-operative bleeding. Rather most of the complications were cardiovascular, respiratory, or urinary in nature.

With close monitoring of the co-morbidities and a programmed operation with a careful pre-operative evaluation and a risk stratication, surgical procedures should be less hazardous, specially in 80 years old patients with an high ASA score.

## Conclusion

Thyroid pathology is particularly important in elderly patients, as the incidence of malignancy increases and they are usually more aggressive tumors. Anaplastic carcinoma presents almost exclusively after the age of 60 years, and differentiated carcinomas are usually more aggressive after the age of 45 years [7], with more frequent extrathyroidal spread and distant metastases [8-10]. Park et al. showed that patients ≥65 years demonstrated more aggressive disease with multiple, larger tumors and more advanced-stage disease, nonpapillary histology, and extrathyroidal extension (p < .001).

The age in itself is not an absolute contraindication for major surgery and few elderly patients receive programmed thyroid surgery due to the major risk of morbidity [1]; some surgeons and endocrinologists prefer to delay the surgical procedure because of the risks of surgery and treat the thyroid disease conservatively with medical or radio-iodine therapy. Common surgical indications in the elderly include hyperthyroidism resistant to medical management, symptoms of compression due to retrosternal goiter extension, suspicion of a malignant nodule requiring histologic diagnosis, or thyroid carcinoma [6,11,12].

It is well known that age plays a fundamental role as a prognostic factor in differentiated thyroid carcinoma [7]. Furthermore, some studies report a higher risk of developing cardiac arrhythmias and osteoporosis in patients older than 60 years who present with a subclinical hyperfunctioning thyroid nodule. For these reasons, a delayed surgical procedure for a suspicious thyroid can expose the elderly patient to increased risks related to subclinical or developed hyperthyroidism, and metastasis in the case of malignancy.

Therefore thyroid surgery in patients aged 70 years or older is safe and the relatively high rate of thyroid carcinoma and toxic goiter may justify an aggressive approach.

We found, within the limits of a retrospective review, that elective thyroid surgery in elderly patients is safe and that age alone should not be a consideration when deciding whether or not to operate. Therefore thyroidectomy at an old age seems to confer a better morbidity and mortality rate when compared with other elective general operative procedures. However, the literature on the outcome of thyroid surgery in the elderly is limited so that randomized studies are necessary to specifically evaluate in homogeneous age groups possible complications and contraindications.

## Competing interests

The authors declare that they have no competing interests.

## Authors’ contributions

RG: conception and design, interpretation of data, given final approval of the version to be published; GO, MAL, BA, GD: acquisition of data, drafting the manuscript, given final approval of the version to be published; AP: conception and design, critical revision, given final approval of the version to be published.
